# Anticipatory Planning in Children with Autism Spectrum Disorder: An Assessment of Independent and Joint Action Tasks

**DOI:** 10.3389/fnint.2016.00029

**Published:** 2016-08-23

**Authors:** Sara M. Scharoun, Pamela J. Bryden

**Affiliations:** ^1^Department of Kinesiology, University of WaterlooWaterloo, ON, Canada; ^2^Department of Kinesiology and Physical Activity, Wilfrid Laurier UniversityWaterloo, ON, Canada

**Keywords:** anticipatory planning, end-state comfort, beginning state comfort, autism spectrum disorder, children

## Abstract

Autism Spectrum Disorder (ASD) is one of the most common neurodevelopmental disorders. Although not a diagnostic feature, motor impairments have been recently acknowledged as prevalent and significant, such that these children have difficulties planning, organizing and coordinating movements. This study aimed to further investigate anticipatory motor planning in children with ASD by means of assessing end- and beginning-state comfort, considering inconsistent reports of end-state comfort in independent action, and the study of beginning-state comfort being limited to one study with young adults. Five- to eleven-year-old children with ASD, and chronologically age- and sex-matched typically-developing children picked-up a glass and: (1) poured a cup of water; and (2) passed it to the researcher to pour a cup of water. End-state comfort was deemed evident if participants grasped the glass thumb-down followed by a 180° rotation; therefore ending with a thumb-up posture. Beginning-state comfort was deemed evident if participants passed the glass to the researcher oriented upright. Findings revealed less end-state comfort in children with ASD, attributed to motor planning deficits. Beginning-state comfort did not differ, ascribed to the habitual nature of the task; therefore reflecting a stimulus-driven response as opposed to an action which reflects anticipatory planning. The findings support difficulties with motor planning and control for children with ASD in an independent task. However, when acting with a familiar object in joint action, behavior does not differ, likely indicative of a habitual, stimulus-driven response.

## Introduction

Epidemiological data from the World Health Organization (2013) estimate that one person in 160 have an Autism Spectrum Disorder (ASD). Diagnosed in childhood and persisting throughout life (American Psychiatric Association, 2013), ASD is a group of neurodevelopmental disorders characterized, in varying degrees, by difficulties with communication, social interaction (i.e., relating to people, things and events), and repetitive behaviors and movements (American Psychiatric Association, 2013). Although not considered a diagnostic feature, sensorimotor impairments have been increasingly acknowledged as prevalent in ASD, and have been shown to significantly impact the quality of life (e.g., Fournier et al., 2010; Gowen and Hamilton, 2013). In particular, individuals with ASD are described as having challenges planning, organizing and coordinating movements (Glazebrook et al., 2008), where a recent review suggests that atypical motor skills result from altered sensory input, deficits in organizing motor knowledge and variable motor execution (Gowen and Hamilton, 2013). This has been well documented in the literature, using reach-to-grasp assessments (e.g., Mari et al., 2003; Sacrey et al., 2014), movement kinematics (e.g., Rinehart et al., 2006), precue paradigms and aiming tasks (e.g., Glazebrook et al., 2008).

One specific challenge in motor planning for individuals with ASD involves the ability to prepare for and anticipate a complete action sequence as opposed to focusing exclusively on the first step (Gowen and Hamilton, 2013). Researchers have thus utilized the end-state comfort effect (Rosenbaum et al., 1990, 2012) as a means of quantifying anticipatory planning behavior; although the breadth of research assessing end-state comfort is limited, and reports are inconsistent (Hughes, 1996; Hamilton et al., 2007; van Swieten et al., 2010; Gowen and Hamilton, 2013). A means to infer evidence of second-order planning, the end-state comfort effect reflects an actor’s consideration of both immediate and subsequent task constraints. For example, when asked to pick up an overturned glass, adults (ages 18–30) are likely to assume an uncomfortable, thumb-down posture at the start of their movement to allow for a comfortable, thumb-up end-state posture, in which the glass is re-oriented for use (Rosenbaum et al., 2012). In comparison to young adults, typically-developing children perform in a manner which highlights a lack of end-state comfort in young children (i.e., ages 3 and 4) that improves with age. It is thus argued that evidence of the effect between ages 5 and 6 is linked to cognitive and sensorimotor development and is not adult-like until approximately age 10 (e.g., Wunsch et al., 2013).

To our knowledge, Hughes (1996) was the first to assess the effect in children with ASD. Using an apparatus which required children (*M*_age (High functioning, *n* = 18)_ = 12.86, *SD* = 2.56; *M*_age (Low functioning, *n* = 18)_ = 13.97, *SD* = 3.95) to pick up from rest and insert one end of a rod (painted half white and half black) into two discs (red and blue) of different diameters, grip selection (comfortable thumb up or awkward thumb down) with the preferred hand was assessed to quantify planning required to complete the task (end-state comfort effect). Results of this study demonstrated that children with autism have problems in executing goal-directed motor acts, as was evident in uncomfortable rod transfers. In other words, children were not sensitive to end-state comfort, but instead displayed a preference for start-state comfort. Therefore, Hughes (1996) suggested children with ASD have planning problems at the level of motor control (Hughes, 1996).

Contrary to Hughes (1996), other researchers have observed no group differences when comparing children with ASD to their typically-developing counterparts. Hamilton et al. (2007; experiment 3) investigated grip selection via a horizontal bar transport task with children with ASD (*M*_age_ = 8 years 1 month, *n* = 25) and typically-developing counterparts. The results revealed the same level of motor planning (i.e., same evidence of end-state comfort) in both groups of children. More recently, van Swieten et al. (2010) explored grip selection in children with ASD (ages 9–14; *M*_age_ = 11.9, *n* = 20) using a modified motor planning task, which required participants to reach and grasp a cylinder and subsequently turn it clockwise or counter clockwise. Children with ASD demonstrated identical performance to age-matched typically-developing children. Here, all 9- to 14-year-olds (children with ASD and typically-developing) and only half of the 5- to 8-year-olds (typically-developing) demonstrated end-state comfort (van Swieten et al., 2010).

Reflecting on the previous, end-state comfort has been used extensively to explore developmental trends within typically-developing and adult populations (Rosenbaum et al., 2012; Wunsch et al., 2013). However, research involving individuals with ASD remains inconclusive (Hughes, 1996; Hamilton et al., 2007; van Swieten et al., 2010), despite reports of planning difficulties with other methods (e.g., Mari et al., 2003; Rinehart et al., 2006; Glazebrook et al., 2008). The current study thus aimed to assess the end-state comfort effect in children with ASD using a modified overturned glass task (Fischman, 1997). Originally used by Fischman (1997), the task involves a drinking glass and a measuring cup filled with water. Participants are asked to pick-up the overturned glass and measuring cup and pour, and pick-up overturned glass, set it down, pick up measuring cup and pour. End-state comfort is evident when participants assumed an uncomfortable thumb-down grasp to start the movement, allowing for a comfortable thumb-up grasp at the end of the movement.

In addition to end-state comfort, recent studies have extended their analyses of the effect to evaluate anticipatory planning during joint interaction (Gonzalez et al., 2011; Ray and Welsh, 2011). Here, adults will typically re-orient an object to facilitate a comfortable grasp (i.e., beginning-state comfort) for a co-actor, while maintaining a comfortable initial grasp (i.e., end-state comfort) for themselves (Gonzalez et al., 2011; Ray and Welsh, 2011). Little research has been conducted to delineate trends in children (Scharoun and Bryden, 2014). In one study, Scharoun and Bryden (2014) observed to demonstrate adult-like patterns of beginning state comfort at the age of 7 in typically-developing children. It was thus argued that children plan for others’ comfort in an adult-like manner earlier than they are able to plan for their own comfort.

To our knowledge, the study of beginning-state comfort in individuals with ASD is limited to one study with adults (Gonzalez et al., 2013). Participants (*M*_age_ = 32.3, *SD* = 11.1, *N* = 10) were presented with a calculator, a toy hammer, and a stick painted half black and half white, where a self-task and other task were utilized. In the self-task, participants were asked to either place or use the tool placed in front of them. In contrast, the other task required the participant to pass the tool to a confederate so he could use or place the tool. In comparison to their typically-developing counterparts, performance of adults with ASD in all of the aforementioned tasks was more variable, thus suggesting different motor planning processes in individuals with ASD (Gonzalez et al., 2013). The current study aimed to assess performance in children with ASD.

Summarizing, the aim of this study was to further elucidate the ability for children with ASD to plan according to end-state comfort, and to assess planning in joint action by means of investigating beginning-state comfort. Research with typically-developing children has noted evidence of adult-like patterns of beginning-state comfort at the age of 7, with end-state comfort following shortly after at the age of 9 (e.g., Scharoun and Bryden, 2014). In individuals with ASD, research has demonstrated inconclusive results with respect to end-state comfort (Hughes, 1996; van Swieten et al., 2010). To our knowledge, this is the first study to investigate beginning-state comfort in children with ASD. Gonzalez et al. (2013) have noted variable performance in adults with ASD. As such, it was hypothesized that, children with ASD would demonstrate less end- and beginning-state comfort than typically-developing counterparts.

## Materials and Methods

### Participants

Fourteen children with ASDs between the ages of 5 and 11 participated in this study (9 male and 5 female). In order to participate, a formal diagnosis of ASD from a medical doctor was required. The Autism Spectrum Quotient: Children’s Version (AQ-Child; Auyeung et al., 2008) was used to quantify autistic traits in children with ASD (see Table 1). The performance of children with ASD was compared to 14 age-, hand preference- and sex-matched typically-developing children who were randomly selected from a larger study that included 75 typically-developing children ages 5–11 (Scharoun and Bryden, 2014). Parents of typically-developing children were not asked to complete the AQ-Child. All children were recruited from summer camps at the institution, and elementary schools in the region. All data were collected outside of the camp or classroom setting. The institution Research Ethics Board approved all recruitment and testing procedures. Written informed consent was obtained from parents/guardians, and assent was obtained from child participants.

**Table 1:** **Demographics of children with Autism Spectrum Disorder (ASD)**.

P#	Age	Sex	HP	AQ
1	5	M	R	90
2	6	F	R	73
3	6	M	R	115
4	7	M	R	109
5	8	F	R	90
6	8	M	R	97
7	8	M	R	113
8	9	F	R	69
9	10	F	L	76
10	10	F	R	107
11	10	M	R	/
12	11	M	R	91
13	11	M	R	114
14	11	M	R	/

*Note that parents of two children did not complete the AQ; however, did have a formal diagnosis of ASD*.

### Procedures and Apparatus

#### Autism Spectrum Quotient: Children’s Version

The AQ-Child (Auyeung et al., 2008) is a 50-item parent questionnaire which was developed to identify autistic traits in 4- to 11-year-old children. Adapted from adult and adolescent versions of the AQ, the AQ-Child takes into consideration five areas linked to autism and the broader phenotype: social skills, attention switching, attention to detail, communication and imagination. A four-point likert scale is used to assess the degree to which parents agree/disagree with statements about their child (0: definitely agree; 1: slightly agree; 2: slightly disagree; and 3: definitely disagree), where items are reverse scored as necessary. Total AQ scores range from 0 (no autistic traits) to 150 (full endorsement on all items), where Auyeung et al. (2008) identified a cut-off score of 76 to have high sensitivity (95%) and specificity (95%). Children with ASD who participated in this study had a range of total scores from 69 to 121, where the mean score was 96.14. Parents of two children did not complete the questionnaire. These children, and two children whose score were below the cut-off score of 76, were not included in analyses which assessed the relationship between AQ-Child scores and measures of anticipatory planning (see below).

#### Overturned Glass Task

**Figure 1:**
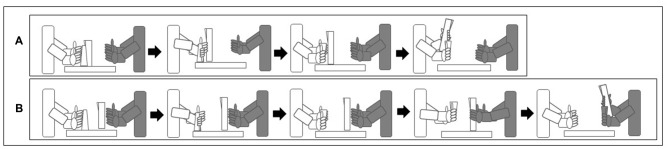
**The participant (white) sat across from the researcher (gray) for the duration of the study.** Participants were asked to complete two different tasks: **(A)** pick-up the glass and pour a cup of water, and **(B)** pick-up the glass and pass it to the researcher to pour a cup of water. Critical trials (overturned glass) are displayed. End-state comfort was deemed evident if participants grasped the glass thumb-down followed by a 180° rotation; therefore ending with a thumb-up posture **(A,B)**. Beginning-state comfort was deemed evident if participants passed the glass to the researcher oriented upright **(B)**.

The apparatus was the same as Scharoun and Bryden (2014). More specifically, the task involved a plastic juice glass (15 cm away from the participant) and a pitcher (filled with two cups of water; 25 cm away from the participant). Neither the glass nor the pitcher had handles. Participants were asked to complete two different tasks: pick-up the glass and pour a cup of water and pick-up the glass and pass it to the researcher to pour a cup of water (see Figure 1). Glass placement was altered between upright (control) and overturned (critical) for each of the tasks, resulting in a total of four separate tasks. Participants completed three trials of each task, for a total of 12 trials.

Previous research has noted that children with ASD demonstrate an insistence on sameness and may demonstrate a resistance to change in the environment (Rutter, 1978). Consequently, a blocked design was implemented, to avoid any potential issues with a randomized design. For example, participants completed all three overturned glass conditions for the bimanual, social task and then transitioned to the three upright glass conditions for the bimanual, social task, and so forth. This was an obvious limitation; therefore, it is suggested that future research assesses whether performance of children with ASD varies based on the order of presentation of trials. Participants were videotaped completing the task, where videos focused on hand movements, but were zoomed out if necessary to capture children’s entire upper limb movements. The first author, who was aware of the purpose of the study, coded videos offline upon completion of the study to denote grasp posture. Only critical trials (i.e., overturned glass) were analyzed to note evidence of end- and beginning-state comfort. End-state comfort was deemed evident if participants grasped the glass thumb-down followed by a 180° rotation; therefore ending with a thumb-up posture. Beginning-state comfort was deemed evident if participants passed the glass to the researcher oriented upright.

## Results

### End-state comfort

**Figure 2:**
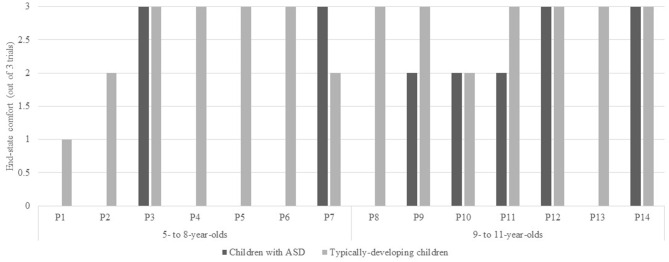
**Number of trials where children with Autism Spectrum Disorder (ASD) and their typically-developing peers displayed end-state comfort.** More specifically, where children grasped the glass thumb-down followed by a 180° rotation; therefore ending with a thumb-up posture.

**Figure 3:**
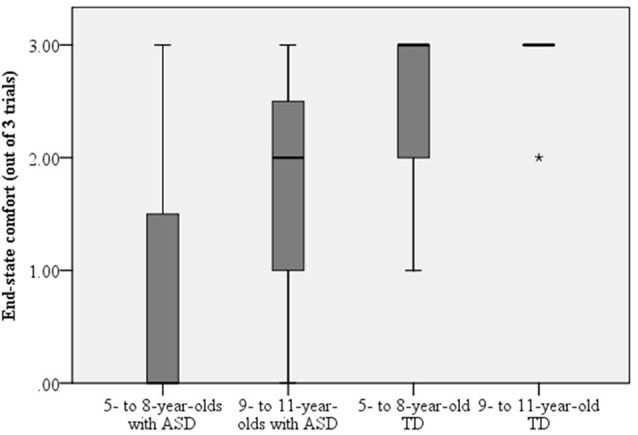
**Evidence of end-state comfort differed between 9- to 11-year-old typically-developing children and 5- to 8-year-olds with ASD, such that typically-developing 9 to 11-year-olds displayed more end-state comfort.** In addition, the comparison between 5- to 8-year-olds with ASD and their typically-developing peers, and between 9- to 11-year-olds with ASD and their typically-developing counterparts were approaching statistically significant differences. Dark lines represent the mean and the *represents an outlier in the data.

Figure 2 depicts the number of trials where children with ASD displayed end-state comfort when grasping the overturned glass to pour a cup of water. A Mann-Whitney test was used to compare end-state comfort in children with ASD and their typically-developing peers. Evidence differed significantly in children with ASD (*Mdn* = 1.00) and their typically-developing peers (*Mdn* = 3.00), *U* = 152.500, *p* = 0.011, *r* = −0.51. Next, a Kruskal-Wallis Test was used to assess end-state comfort when children with ASD and their typically-developing peers were separated into younger (i.e., ages 5–8) and older (i.e., ages 9–11) groups. End-state comfort was significantly affected by group, *H*_(3)_ = 8.68, *p* = 0.034 (see Figure 3). Pairwise comparisons with adjusted *p*-values showed that there were no significant differences between 5- to 8- and 9- to 11-year-olds with ASD (*p* = 0.428, *r* = −0.15), 9- to 11-year-olds with ASD and 5- to 8-year-old typically-developing children (*p* = 0.267, *r* = −0.21), and 5- to 8- and 9- to 11-year-old typically-developing children (*p* = 0.408, *r* = −0.16). There was a significant difference between 5- to 8-year-olds with ASD and 9- to 11-year-old typically-developing children (*p* = 0.006, *r* = −0.52). Comparisons between 5- to 8-year-olds with ASD and 5- to 8-year-old typically-developing children (*p* = 0.057, *r* = −0.36), and 9- to 11-year-olds with ASD and 9- to 11-year-old typically-developing children (*p* = 0.053, *r* = −0.37) were approaching statistically significant differences.

### Beginning-State Comfort

**Figure 4:**
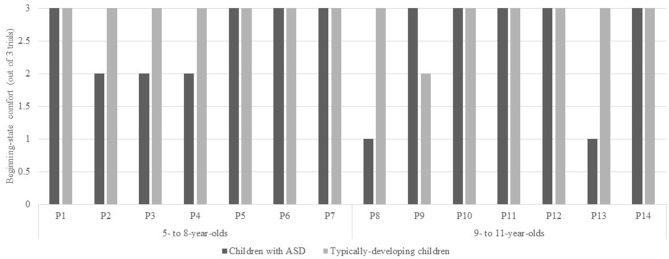
**Number of trials where children with ASD and their typically-developing peers displayed beginning-state comfort.** More specifically, when participants passed the glass to the researcher oriented upright.

**Figure 5:**
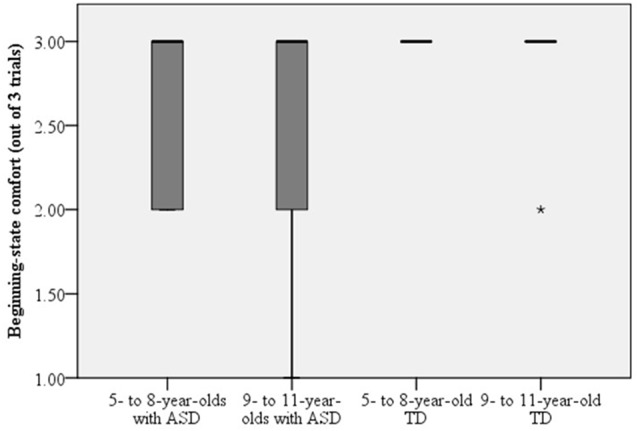
**Beginning-state comfort did not differ between children with ASD or typically developing children regardless of age.** Dark lines represent the mean and the *represents an outlier in the data.

The number of trials where children with ASD displayed beginning-state comfort is found in Figure 4. A Mann-Whitney test was used to compare beginning-state comfort in children with ASD and their typically-developing peers. The evidence did not differ significantly in children with ASD (*Mdn* = 1.00) and their typically-developing peers (*Mdn* = 3.00), *U* = 69.00, *p* = 0.063, *r* = −0.35. Next, a Kruskal-Wallis Test was used to assess end-state comfort when children with ASD and their typically-developing peers were separated into younger (i.e., ages 5–8) and older (i.e., ages 9–11) groups. Beginning-state comfort was not affected by group, *H*_(3)_ = 3.92, *p* = 0.271 (see Figure 5).

### Relationship Between Measures of Anticipatory Planning and AQ-Child Scores

**Figure 6:**
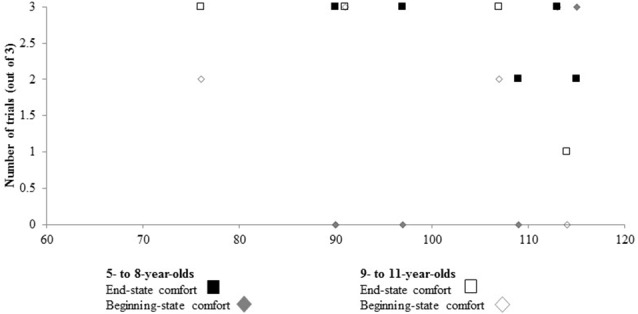
**No relationship emerged between Autism Spectrum Quotient: Children’s Version (AQ-Child) scores and ESC or BSC when collapsed as a function of age, or when separated into age groups.** Note that the horizontal axis starts at a score of 60.

Pearson correlations were used to assess the relationship between AQ-child scores and measures of anticipatory planning. No significant relationships emerged between AQ-child scores and end-state comfort or beginning-state comfort (see Table 2 and Figure 6) overall or when separated into younger (ages 5–8) and older groups (ages 9–11).

**Table 2:** **Correlations between Autism Spectrum Quotient: Children’s Version (AQ-Child) scores and measures of anticipatory planning (end-state comfort and beginning-state comfort)**.

	End-state comfort	Beginning-state comfort
Total	*r* = 0.120, *p* = 0.740	*r* = −0.613, *p* = 0.060
5- to 8-year-olds	*r* = 0.712, *p* = 0.073	*r* = −0.053, *p* = 0.911
9- to 11-year-olds	*r* = −0.008, *p* = 0.990	*r* = −0.005, *p* = 0.994

## Discussion

This study aimed to delineate the ability for children with ASD to plan according to end- and beginning-state comfort. Based on previous research (e.g., Gonzalez et al., 2013; Gowen and Hamilton, 2013), it was hypothesized that children with ASD would demonstrate less end- and beginning-state comfort than typically-developing peers. The following sections will discuss results pertaining to similarities and differences that emerged.

### End-State Comfort

The first stage of analyses compared evidence of end-state comfort in children with ASD and their typically-developing peers. In line with our hypothesis, a significant difference emerged, such that children with ASD displayed less end-state comfort. To examine this further, children were separated into younger (ages 5–8) and older (ages 9–11) groups, based on previous reports of adult-like patterns in typically-developing children at approximately ages 9 or 10 (e.g., Wunsch et al., 2013). Five to eight-year-olds with ASD displayed less end-state comfort than 9- to 11-year-old typically-developing children. Furthermore, comparisons between 5- to 8-year-olds with ASD and 5- to 8-year-old typically-developing children (*p* = 0.057), and 9- to 11-year-olds with ASD and 9- to 11-year-old typically-developing children (*p* = 0.053) were close to the benchmark (*p* < 0.05) typically used to infer a significant difference. In line with our hypothesis, children with ASD displayed less end-state comfort. These results are contrary to Hamilton et al. (2007) and van Swieten et al. (2010), who observed identical performance in age-matched children with ASD and typically-developing children. Findings thus provide support for Hughes (1996) who suggested planning problems exists in children with ASD at the level of motor control.

Motor planning in object manipulation is challenging, because it requires consideration of the entire movement sequence, as opposed to simply the first step (Gowen and Hamilton, 2013); therefore, an action plan must be created before the action is completed (Manoel and Moreira, 2005; Adalbjornsson et al., 2008). Research has noted that tasks involving action chaining—movements linked in overlapping segments (Graybiel, 1998; Gobet et al., 2001)—are challenging for children with ASD; therefore, these children are observed performing each element of the task individually. As evidenced in the current study, rather than planning for secondary task requirements (i.e., object use), children with ASD displayed behaviors indicative of first-order planning (i.e., immediate reach-to-grasp; Rosenbaum et al., 2012); therefore displaying start-state comfort as opposed to end-state comfort.

Hughes (1996) referred to deficits in sequencing abilities, further explaining how impairment in sequential activity is not limited to, multiple movements, but can be observed in single purposeful movements as well. Hughes (1996) also emphasized the importance of prediction in movement (Poulton, 1957), such that children with ASD are restricted in their understanding of the consequences associated with one’s own actions as they pertain to the decision to plan that action. Finally, once an action plan is formulated, it is both difficult and costly for children with ASD to deviate from the original plan (Hill, 2004). As evidenced in the current study, difficulties planning, organizing and coordinating movements emerge when children with ASD are challenged to plan according to end-state comfort (Glazebrook et al., 2008; Gowen and Hamilton, 2013).

In comparison to Hughes (1996), and van Swieten et al. (2010) observed that the majority of 9- to 14-year-olds with and without ASD displayed end-state comfort. These results are in line with recent reports of typical development, where end-state comfort was comparable to adults by age 9 (Stöckel et al., 2012; Scharoun and Bryden, 2014). With respect to Hughes (1996) findings, van Swieten et al. (2010) hypothesized that differences in findings are likely attributable to experimental design. More specifically, van Swieten et al. (2010) had participants grasp and rotate a cylinder either clockwise or counterclockwise, whereas Hughes (1996) task required participants to grasp a rod and transport it to a disc so it stood upright. It was argued that Hughes (1996) task was more complex; therefore, planning deficits emerged in children with ASD (van Swieten et al., 2010; see also Sacrey et al., 2014).

In the present study, children were required to grasp the overturned glass and the pitcher in anticipation of pouring water. It is thus likely that the bimanual nature of the task increased complexity. Compared to a unimanual task, more extensive cognitive processing and motor planning are required in a bimanual task (Logan and Fischman, 2011). Bimanual actions require an interplay between intra- and inter-limb coordination (Bobbio et al., 2009), while simultaneously processing multisensory information from the environment (Brakke et al., 2007). Therefore, it is likely that the increased task complexity in the current study led to findings similar to Hughes (1996). Nevertheless, it is also important to note that Hamilton et al. (2007) implemented a bar-transport task similar to Hughes (1996), but failed to replicate the findings. Recent work with typically-developing children comparing object manipulation tasks note end-state comfort emerges earlier in the overturned glass task compared to the bar-transport task (Knudsen et al., 2012). Future work assessing the effect in children with ASD would benefit from the comparison of multiple tasks, to identify the nature of inconsistent results.

### Beginning-State Comfort

Unlike the differences that emerged between the groups in the assessment of end-state comfort, when asked to pick-up the overturned cup and pass it to the researcher to pour water, behavior of children with ASD did not differ from their typically-developing peers. This was surprising, considering it is suggested that impaired motor planning skills may impede a child’s ability to investigate their physical and social environment (Glazebrook et al., 2008). Furthermore, deficits in social-motor behaviors have been linked to malfunctioning of the mirror neuron system (Williams et al., 2004); where the autistic mirror neuron dysfunction hypothesis predicts behaviors such as goal inference, to be atypical in children with ASD (Hamilton et al., 2007).

Despite evident difficulties in social environments, behaviors in children with ASD—communication skills (Bernard-Opitz, 1982), physical and eye contact (Kasari et al., 1993) and social interaction (Knott et al., 1995)—have been shown to improve during interactions with familiar people (parent, sibling) in comparison to a stranger or peer. As such, it is suggested that dysfunctions in the mirror neuron system may be resultant of an underlying deficit in the ability to identify with unfamiliar things (Oberman et al., 2008). Furthermore, it is suggested that, in contrast to typically-developing children who identify all things (familiar and unfamiliar) as socially congruent, children with ASD may only denote familiar things to be socially compatible (Oberman et al., 2008).

Observing the familiarity of the current task, previous studies investigating the development of feeding behaviors suggest that children slowly gain experience with cups prior to their second birthday. Therefore, cup-use is a familiar action for children (Carruth and Skinner, 2002), where certain habitual objects, are perceived to afford specific functional movements (Herbort and Butz, 2011). As such, it is likely that children with ASD were displaying a habitual response, as opposed to an action indicative of motor planning. Future research with other objects is necessary to confirm or refute this hypothesis.

### Relationship Between Measures of Anticipatory Planning and AQ-Child Scores

Interestingly, no relationship emerged between measures of anticipatory planning (i.e., end- and beginning-state comfort) and AQ-child scores. This it likely attributed to the heterogeneity of ASD that is commonly reported in the literature, genetically, neurologically, and behaviorally (e.g., Happé et al., 2006; Jeste and Geschwind, 2014). With respect to motor behavior, it has been argued that a better understanding of such processes will enable the heterogeneity of ASD to be disentangled (e.g., Gowen and Hamilton, 2013). As such, continued research in this area is of utmost importance.

### Summary and Conclusion

Recent investigations have noted children with ASD perform identical to their typically-developing counterparts in motor planning tasks (Hamilton et al., 2007; van Swieten et al., 2010). However, planning problems at the level of motor control (e.g., Hughes, 1996) have led to motor impairments being identified as a cardinal feature of ASD (Fournier et al., 2010; Gowen and Hamilton, 2013). Although the literature is inconclusive, it was hypothesized that children with ASD would demonstrate significantly less end- and beginning-state comfort than their typically-developing counterparts. The results of this study are in agreement with our hypothesis with respect to end-state comfort; however, no differences emerged in the assessment of beginning-state comfort. The findings support the notion that children with ASD have difficulties in planning and controlling movement in independent task. However, when acting with a familiar object in joint action, behavior does not differ, likely indicative of a habitual response as opposed to a planned behavior.

### Implications and Future Directions

It is well known that “neurological diseases exact an exorbitant health cost on our population” (Ajemian and Hogan, 2010, p. 337). Furthermore, other aspects of life—social (Gray, 1993), health (Allik et al., 2006) and overall life/lifestyle (Sen and Yurtsever, 2007)—of families and caregivers (Fletcher et al., 2012) of children with ASD are negatively affected. In order to mitigate these costs, we must develop a better understanding of the neurological foundations of these diseases (Ajemian and Hogan, 2010). It is suggested that motor symptoms may play a larger role in autism than originally believed. Therefore, transitioning towards a movement perspective may have implications for the development of new diagnostic criteria and intervention protocol (Leary and Hill, 1996; Provost et al., 2007), as recent investigations have suggested motor impairments are a cardinal feature of ASD (Fournier et al., 2010). Furthermore, in order to provide the best services for children with ASD, therapists must be aware of motor difficulties as they pertain to motor control (Mari et al., 2003; Provost et al., 2007); thus highlighting the importance of current and future research studies, which aim to delineate motor difficulties in children with ASD.

## Author Contributions

SMS completed this work as part of her MSc thesis. She was responsible for study design, data collection, analyses and interpretation and writing of the manuscript. PJB supervised the thesis, and was thus also responsible for study design, analyses, interpretation and writing of the manuscript.

## Conflict of Interest Statement

The authors declare that the research was conducted in the absence of any commercial or financial relationships that could be construed as a potential conflict of interest.
